# Experimental robustness and reproducibility of the murine cecal ligation and puncture sepsis model

**DOI:** 10.1186/s40635-026-00950-0

**Published:** 2026-07-14

**Authors:** Korbinian Felix Schreyer, Julia Stettmer, Li Cheng Wu, Markus Ballmann, Johannes Krell, Gerhard Schneider, Martin Schlegel

**Affiliations:** Department of Anesthesiology and Intensive Care, School of Medicine and Health, Technical University of Munich, Munich, Germany

**Keywords:** Sepsis, Animal models, Cecal ligation and puncture (CLP), Sex differences, Immune response, Hematopoiesis, Reproducibility, Standardization

## Abstract

**Background:**

Sepsis, a dysregulated host response to infection leading to organ failure, remains a leading cause of death worldwide, reflecting the failure to translate preclinical findings into clinical therapies. This translational gap is partly driven by substantial variability and limited reproducibility of preclinical models, particularly the cecal ligation and puncture (CLP) model, widely regarded as the gold standard among experimental sepsis models. Although multiple biological and methodological variables influence outcomes, their relative contributions remain incompletely defined, and standardized approaches for integrated immune and hematopoietic profiling are lacking.

**Methods:**

We established and validated a standardized experimental and analytical framework for CLP-induced sepsis, enabling controlled disease severity and integrated assessment of local and systemic immune responses. A total of 88 C57BL/6 mice underwent mid-grade sepsis (target survival ~ 70%) across 19 independent experiments performed by different operator teams. The primary endpoint was time to death or predefined humane endpoints within 48 h, aiming to identify biological and experimental variables influencing survival and immune responses. Immune cell populations, hematopoietic stem and progenitor cell subsets, and cytokines were quantified by multiparameter flow cytometry, and tissue responses were assessed histologically. Survival dynamics and the influencing variables were analyzed using Kaplan–Meier estimates and Cox proportional hazards regression.

**Results:**

The framework yielded consistent mid-grade sepsis, with a 48 h survival rate of 67.2% (95% CI 54.6–78.2%). Survival outcomes and immune readouts were reproducible across operators and experiments, demonstrating robust model performance under standardized conditions. Among tested variables in a multivariate Cox regression analysis, experiments performed during summer were associated with increased mortality (HR 3.178, 95% CI 1.164–8.681, *P* = 0.024), whereas sex, age, body weight, day time, and cage density had no significant effect on survival (all *P* > 0.05). Integrated immune and hematopoietic profiling revealed sex-dependent immune responses that were not associated with altered survival.

**Conclusions:**

This standardized CLP framework enables reproducible survival outcomes that are largely independent of common biological and procedural variables. Consistent results can be obtained across approximately three independent experiments, each including around 8 animals per group. Systematic assessment of biological and environmental variability may improve reproducibility and support more consistent interpretation of CLP-based sepsis studies.

**Supplementary Information:**

The online version contains supplementary material available at 10.1186/s40635-026-00950-0.

## Background

Sepsis, as defined by the Sepsis-3 guidelines, is characterized by a dysregulated host immune response resulting in life-threatening organ dysfunction [1]. The cecal ligation and puncture (CLP) mouse model is the most widely used preclinical sepsis model and often referred to as the gold standard [2, 3] because it mimics the clinical progression of human sepsis, including polymicrobial infection and multiple organ failure [4]. Despite its widespread use, sepsis survival rates in the CLP model vary significantly across laboratories, depending strongly on the portion of the caecum ligated, with some reporting rates of 20% to 50% [3, 5, 6]. Insufficient standardization, or rather, as suggested by some, the lack of systematic heterogenization, and resulting inter-study variability are major contributors to the limited translational success of murine sepsis models, alongside fundamental biological and clinical differences between experimental systems and human disease [7–9].

Heterogeneity in animal models simulating human diseases remains a major obstacle in translational research. This variability is driven by procedural inconsistencies, lack of standardization or systematic heterogenization, environmental factors, experimenter-dependent effects, and sex-specific biological differences, and has been reported in various pathologies, inter alia, in liver disease, bone regeneration, subarachnoid hemorrhage, and autism spectrum disorder [10–14]. In the murine CLP sepsis model, procedural determinants of disease severity are relatively well characterized, whereas broader biological, environmental, and experimenter-related sources of variability have been investigated more selectively and remain comparatively undercharacterized [15–17].

In addition to peripheral immune responses, sepsis induces profound alterations in hematopoiesis, including emergency myelopoiesis, hematopoietic stem and progenitor cell (HSPC) dysfunction, impaired lymphopoiesis, and remodeling of the bone marrow niche [18–22]. Although hematopoietic remodeling is increasingly recognized as a relevant component of sepsis pathophysiology, bone marrow and HSPC responses remain insufficiently characterized and are not routinely integrated into CLP study designs. In addition, the lack of standardized hematopoietic analyses and sex-stratified reporting within current preclinical sepsis frameworks substantially limits comparability and mechanistic interpretation across studies.

Survival and secondary outcomes in rodent experiments depend on a wide range of factors beyond the disease model itself. The rodent strain has been described as a significant determinant of survival, inter alia, in the CLP model [23] and in a model of melioidosis [24]. In a model investigating corticosteroid-induced side effects, even the mouse substrain significantly altered survival and the severity of side effects [25]. Moreover, age [26, 27], alterations in the microbiome [28], diets [29–31], and time of hit [32] might alter the course of the disease and its outcome. Additionally, the therapeutic application of fluids, antibiotics [33], or analgesics [34, 35] remains inconsistent and is often not reported [3]. However, independent factors such as body weight, facility hygiene standards, and cage density [36, 37] are less well defined. Mice are usually housed in groups, limiting space consumption and costs, and increased cage density has repeatedly not been linked to reduced well-being [38]. Their impact on sepsis outcome remains unknown.

Therefore, we aimed to establish a standardized CLP workflow that enables controlled disease severity, integrated local, systemic, and hematopoietic immune profiling, and systematic assessment of biological, environmental, and procedural variables affecting reproducibility. We analyzed key biological variables (sex, age, body weight) alongside environmental and technical factors (surgeon, season, cage density, animals per surgeon). Systematic assessment and reporting of these variables may help to improve the reproducibility and interpretation of preclinical sepsis research.

## Results

### Workflow

**Fig. 1:**
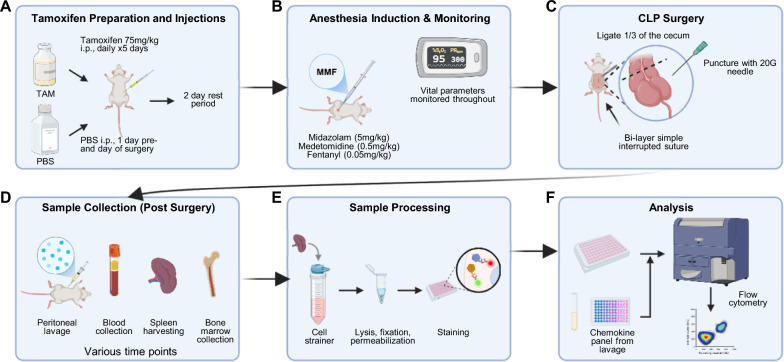
Experimental workflow.** A**, Mice were injected with tamoxifen once daily for five consecutive days, followed by a two-day resting period, or received PBS. **B**, Anesthesia was induced using midazolam, medetomidine, and fentanyl (MMF), and vital parameters were monitored. **C**, During CLP, one third of the cecum was ligated and punctured once inside-out with a 20G needle. **D**, Peritoneal lavage, peritoneum, blood, spleens, bones, and tail tips were harvested. **E**, Spleens and bone marrow were passed through a cell strainer, erythrocytes were lysed, and cells in single cell suspensions were fixed, permeabilized, and stained. **F**, Stained single cells and chemokines bound to pre-coated beads were acquired on a BD LSRFortessa™ flow cytometer. Created in BioRender. For citation, see the methods section

To provide a consistent experimental and analytical workflow (Fig. 1), we implemented a standardized, reproducible protocol that enables controlled modulation of disease severity and integrates multi-compartment immune monitoring in CLP-induced sepsis. While the CLP model has been established more than 40 years ago as a clinically relevant model of polymicrobial sepsis [39], we aimed to dissect factors impacting its reliability and reproducibility of not only the survival, but also the elicited immune response, under standardized conditions. The workflow begins with 5 days of consecutive tamoxifen (75 mg/kg BW (body weight)) intraperitoneal administration to induce gene deletion in genetically modified mice, alongside corresponding control animals (Fig. 1A). Only the latter, which are phenotypically wild-type, are part of this analysis. After 2d of recovery, mid-grade CLP sepsis was induced by ligation of one third of the cecum, inside-out puncture of the cecum using a 20G needle, and gentle extrusion of cecal contents (Fig. 1B–C). At predefined time points (12 h, 24 h, 48 h, and 96 h), local immune cells from the peritoneal cavity and systemic circulation were collected, along with tissues including peritoneum, spleen, and bone marrow (Fig. 1D). For survival analyses, 48-h survival was defined as the primary endpoint, as the majority of animals were observed within this time window, ensuring a consistent and comparable assessment across experimental cohorts. Animals that survived beyond 48 h (n = 8) were administratively right-censored at 48 h for the purpose of the Kaplan–Meier analysis. Downstream analyses integrated cellular, soluble, and tissue-level readouts, with immune cell populations and cytokines quantified by multiparameter flow cytometry and tissue responses assessed histologically for leukocyte infiltration and local inflammation (Fig. 1E–F). Survival outcomes and the influence of biological and experimental variables were analyzed using Kaplan–Meier estimates, univariate Cox proportional hazards models for the individual variables, and a multivariate Cox proportional hazards model for the combined variables as covariates. All procedures were performed according to predefined standard operating procedures to ensure consistency and reproducibility across experiments and operators. A detailed stepwise description of the protocol is provided in the Methods section, and representative images illustrating the CLP procedure are shown in Supplementary Figure S1.

### Baseline cohort characteristics and model performance

**Fig. 2:**
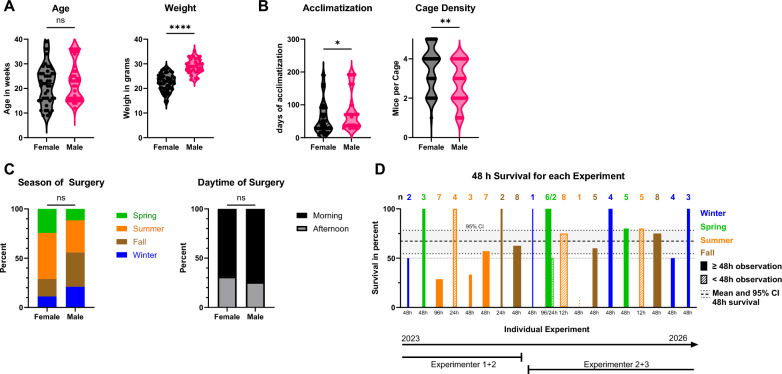
Baseline parameters. **A**, Violin plots display the age and baseline body weight of female (black) and male (red) mice. **B**, Violin plots of acclimatization time in days and cage density in mice per cage of female (black) and male (red) mice. **C**, Stacked bars plot of the season and daytime of surgery. **D**, Bars plot of the 48 h survival in each of the 19 individual experiments, with the width corresponding to the number of animals as displayed above the column. Mean survival (bold dotted line) and 95% CI (dotted lines) are shown for experiments lasting at least 48 h. Experiments with a shorter observation period are indicated by hatched bars. The observation time for each experiment is noted below the corresponding bar. The bar for Experiment 10 is divided into two segments, as a subset of animals was followed for up to 24 h, whereas the remaining animals were observed for up to 96 h. Violin plots in **A-B** with median and Kernel Density. Welch test (**A** weight graph), Mann–Whitney test (**A** age graph, **B**), Chi-square test (**C**). *P < 0.05, **P < 0.001, ****P < 0.0001

To assess the robustness and reproducibility of the experimental system, we analyzed 88 phenotypically wild-type animals (43 males, 45 females) subjected to mid-grade CLP-induced sepsis between 2023 and 2026. Animals varied in age from 9 to 40 weeks and in body weight from 14.7 to 35 g, with sex-specific distributions detailed in Table 1, Fig. 2A–B, and Figure S2A–B. The majority of animals (92%) received tamoxifen pretreatment prior to CLP induction. Experiments were conducted in 19 independent runs by three operators, with acclimatization periods ranging from 7 to 200 days following transfer from an external facility (Fig. 2B–D). Animals were followed for up to 96 h, with predefined harvesting time points at 12 h (14.8%), 24 h (9.1%), 48 h (61.4%), and 96 h (14.8%). Of the 67 mice with follow-up intervals of at least 48 h, 45 animals survived the first 48 h (67.2%, 95% CI 54.6–78.2%), corresponding to the intended mid-grade disease severity. Mortality occurred in a controlled temporal pattern, with no excessive early deaths and a consistent time-to-event distribution over the observation period. No median survival time could be computed, as more than 50% of animals survived the observation period.. No operator-team-dependent differences in survival were observed (*P* > 0.05). Survival rates varied slightly between experiments; however, in a binomial logistic regression, no individual experiment with a postoperative observation period of at least 48 h differed significantly from the reference regarding 48 h survival (all *P* > 0.05) (Fig. 2D). Together, these data demonstrate a stable, reproducible model performance across operators, independent experiments, and time, despite controlled biological and experimental variability, providing a robust basis for systematically assessing the influence of these factors on survival and immune responses.

**Table 1:** Baseline characteristics

Variable	Female Mice	Male Mice	All Mice	*P* (female vs male)
Number, n	45	43	88	
48 h Mortality, n (%)	14 (31.1)	12 (27.9)	26 (29.5)	0.742^1^
48 h Mortality in mice with Follow-up ≥ 48 h	11 (33.3)	11 (32.4)	22 (32.8)	0.932^1^
Age, weeks, median (IQR)	22 (11)	23 (12)	22.5 (12)	0.864^2^
Body Weight, gram, mean ± SD	21.7 ± 3.1	28.8 ± 2.8	25.1 ± 4.6	** < 0.0001**^**3**^
Follow-up				0.388^1^
12 h, n (%)	6 (13.3)	7 (16.3)	13 (14.8)	
24 h, n (%)	6 (13.3)	2 (4.7)	8 (9.1)	
48 h, n (%)	25 (55.6)	29 (67.4)	54 (61.4)	
96 h, n (%)	8 (17.8)	5 (11.6)	13 (14.8)	
Cage density, animals per cage, median (IQR)	4 (2)	3 (2)	3 (2)	**0.002**^**2**^
Animals per experimenter, median (IQR)	7 (1.8)	6 (2.0)	7 (2.0)	0.868^2^
Acclimatization, days, median (IQR)	38 (63)	51 (35)	48 (62)	0.216^2^
Daytime of surgery				0.565^1^
Morning, n (%)	31(68.9)	32 (74.4)	63 (71.6)	
Afternoon, n (%)	14 (31.1)	11 (25.6)	25 (28.4)	
Season of surgery				0.076^1^
Spring, n (%)	11 (24.4)	5 (11.6)	16 (18.2)	
Summer, n (%)	21 (46.7)	14 (32.6)	35 (39.8)	
Fall, n (%)	8 (17.8)	15 (34.9)	23 (26.1)	
Winter, n (%)	5 (11.1)	9 (20.9)	14 (15.9)	
Oat supplementation, n (%)	24 (53.3)	24 (55.8)	48 (54.5)	0.815^1^
TAM injection, n (%)	45 (100)	36 (83.7)	81 (92.0)	**0.005**^**1**^

Normally distributed data are presented as mean ± standard deviation (SD); non-normally distributed data are presented as median (interquartile range, IQR). Superscript numbers indicate the statistical test used. ^1^chi-square (χ^2^) test, ^2^Mann-Whitney U test, ^3^Welch test. Significant *P*-values are in bold letters.

### Biological and experimental determinants of survival

**Fig. 3:**
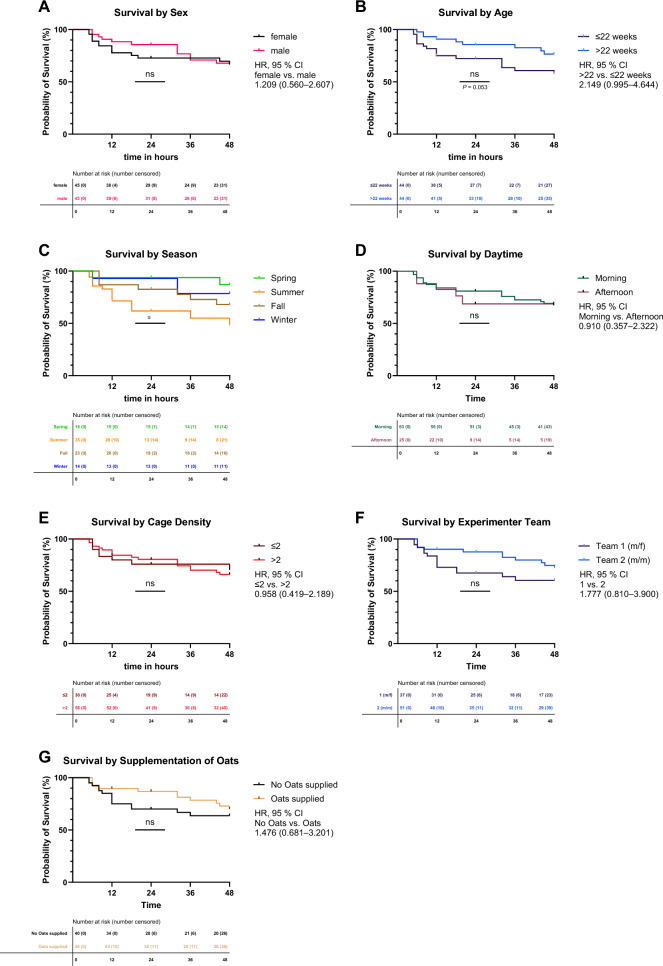
Survival in the Experimental Cohort. Survival depending on **A** the sex, **B** the age, **C** the season, **D** the daytime, **E** the cage density, **F** the experimenter team and their sex, **G** the supplementation of oats. Survival was estimated using the Kaplan–Meier method. Statistical significance refers to the log-rank test. Hazard ratios were derived from univariate Cox proportional hazards regression. Censored events are indicated by tick marks. *P < 0.05

To determine which variables affected survival, the most commonly assessed primary outcome, we investigated the influence of sex, age, season, cage density, experimenter team, and the presence or absence of oat supplementation. Each variable was analyzed independently using univariable Cox proportional hazards regression analysis. Sex of the mice had no significant effect on survival (Fig. 3A). We divided the animals into two age cohorts of equal size (age ≤ 22 weeks or > 22 weeks; n = 44 per cohort). Sex distribution did not differ between the age groups (no expected cell frequencies were below 5, χ^2^(1) = 0.045, *P* = 0.831) (Suppl. Figure 3A), though did seasonal distribution among age groups (two expected cell frequencies were < 5, Fisher’s exact test *P* < 0.0001) (Suppl. Figure 3B). Surprisingly, older mice displayed a non-significant higher probability of survival (HR 2.149, 95% CI 0.995–4.644, *P* = 0.054) (Fig. 3B). As previously reported [16], survival differed across seasons, presenting lower survival during summer (*P* = 0.031; Fig. 3C, for individual experiments see Fig. 2D). In a direct comparison of summer versus the combined other seasons, survival was significantly decreased during summer (HR 2.73, 95% CI 1.18–6.32, *P* = 0.0058; Suppl. Figure 3C). As sex and season were not evenly distributed, the seasonal association should be interpreted as hypothesis-generating rather than causal. There was no noticeable effect of the time of the day of the surgery (*P* = 0.834) (Fig. 3D). Cage density had no effect on survival (*P* = 0.919) (Fig. 3E). Mice cared for by team 1 (male scientists only) compared to team 2 (mixed sex) showed a slightly higher probability of survival in the early phase of the experiment; however, this difference diminished over time and was not statistically significant (*P* = 0.133; Fig. 3F). When oats were supplemented on the cage floor perioperatively, mice showed a slightly improved survival during the early phase after sepsis induction, although this difference was not statistically significant (*P* = 0.312; Fig. 3G). Similarly, mice receiving oats exhibited somewhat less weight loss during the first 12 h after sepsis induction (*P* = 0.114) (Suppl. Figure 3D). Tamoxifen pretreatment showed no detectable association with survival (*P* = 0.721; Suppl. Figure 3E) or peritoneal immune cell migration (Suppl. Figure 3F). In this cohort, however, the untreated comparison group was small (n = 7), vastly limiting definitive conclusions.

### Adjusted effects on sepsis survival

**Fig. 4:**
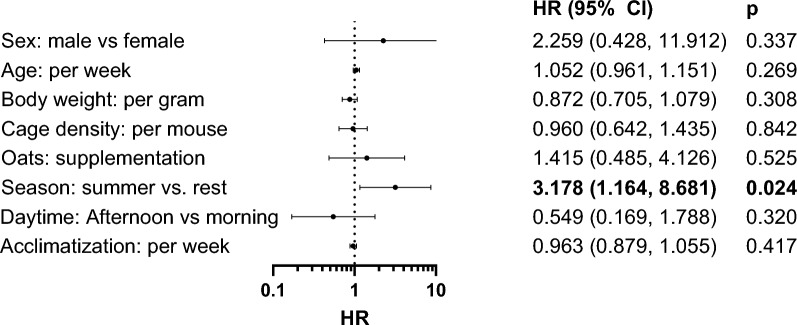
Forest plot of hazard ratios from multivariate Cox proportional hazards regression analysis. Hazard ratios with 95% confidence intervals for all variables included in the model are displayed. Significant hazard ratios and *P*—values are in bold letters

To evaluate the combined influence of multiple covariates on sepsis survival, we employed a multivariate Cox proportional hazards regression model to estimate their individual and joint effects. The model included sex, age, body weight, season (summer vs. other), daytime (afternoon vs. morning), oat supplementation, and cage density as covariate predictors of 48 h survival. The overall model, representing the combined effects of all included factors, did not achieve statistical significance (Omnibus test, *P* = 0.189). Only the summer season showed a significant negative effect on survival (HR 3.178, 95% CI 1.164–8.681, *P* = 0.024) (Fig. 4).

### Profiling the local intraperitoneal immune cells

**Fig. 5:**
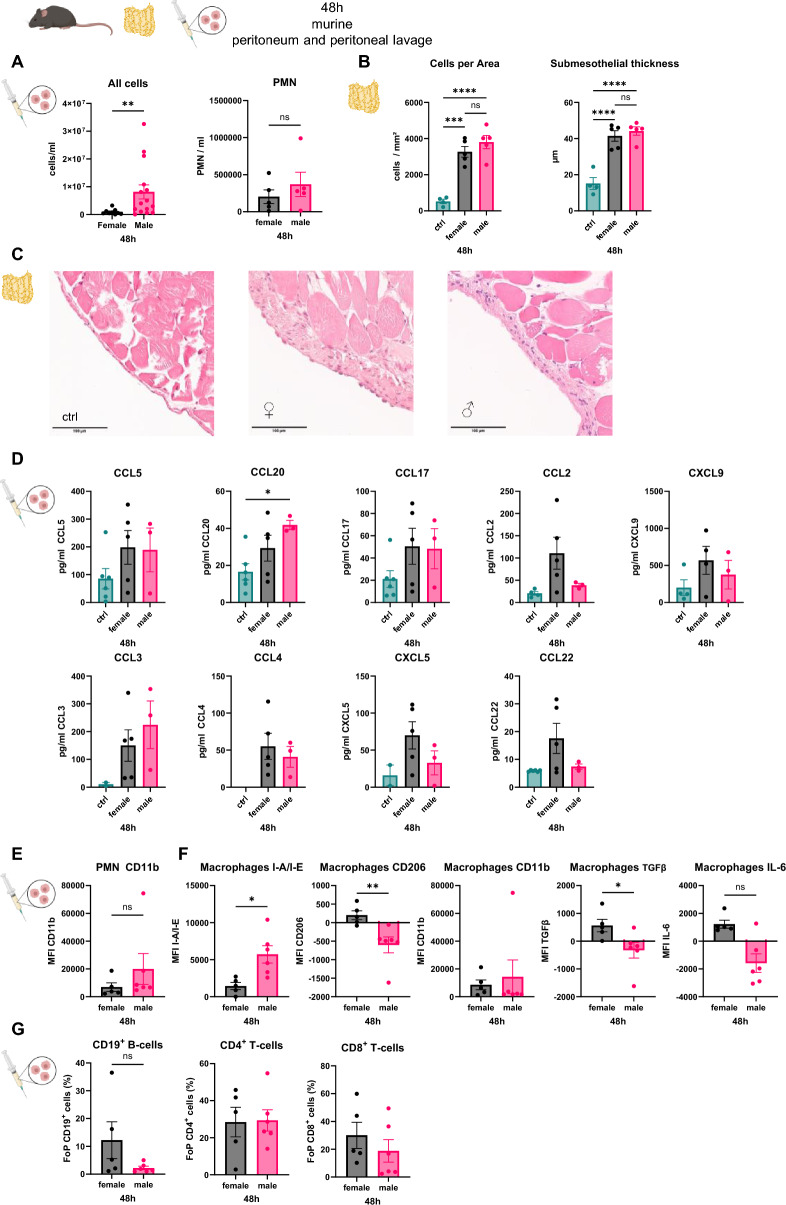
Sex-dependent differences of the intraperitoneal immune response*.*** A**, Total cell counts and PMNs within the peritoneal lavage at 48 h after sepsis induction in female (black) and male (red) mice**. B**, Local peritoneal inflammation was assessed by H&E staining of the peritoneum. Subserosal cellular infiltration and submesothelial thickness were quantified in baseline healthy control (cyan), female (black), and male (red) mice at 48 h after sepsis induction. **C**, We obtained representative, HE-stained sections of baseline healthy control (left), female (middle), and male (right) mice after 48 h of sepsis. **D**, Chemokines within the peritoneal lavage in baseline healthy control (cyan), female (black), and male (red) mice after 48 h of sepsis were assessed using a multiplex flow cytometry chemokine panel. Expression of surface markers and cytokines in neutrophils (**E**) and macrophages (**F**) after 48 h of sepsis in female (black) and male (red) mice is displayed as MFI. **G**, We determined T- and B-cell composition in the peritoneal lavage of female (black) and male (red) mice. Data are mean ± SEM, *P*-values were determined by Mann–Whitney test (**A** Lavage, **D**, **E**, except I-A/I-E), Welch test (**A** PMN, **E** I-A/I-E), respectively 1-way ANOVA with post hoc Tukey test (**A** and **C**). *P < 0.05, **P < 0.01, ***P < 0.001, ****P < 0.0001. Partially created in BioRender

To characterize sex-dependent effects in the local intraperitoneal immune response, we assessed innate and adaptive immune cells, cytokines in the peritoneal lavage, and histological features of HE-stained peritoneal sections at 48 h. Male mice displayed a significantly increased leukocytes influx into the peritoneal cavity compared to females (median cell counts 4.02 × 10^6^ vs 5.58 × 10^5^ cells/ml, *P* = 0.0054), including a trend for higher neutrophil counts (median cell counts 2.78 × 10^5^ vs 1.22 × 10^5^ cells/ml, *P* = 0.4091) (Fig. 5A). During the summer season, cell influx into the peritoneal cavity was significantly lower (median cell counts 4.74 × 10^5^ vs 3.11 × 10^6^ cells/ml, p = 0.0072) (Suppl. Figure 4). However, it should be again noted that sex and season were not evenly distributed in the experimental groups. In contrast, in peritoneal sections, no difference in cells per area in the subserosa (*P* = 0.415) and submesothelial thickness (*P* = 0.491) (Fig. 5B + C) was observed. Intraperitoneal chemokine levels revealed only minor sex-dependent (Fig. 5D) or season-dependent (Suppl. Figure 4) differences. Functional analysis of immune cells indicated a tendency towards M2 macrophage polarization and elevated TGF-β levels in females, accompanied by higher B cell numbers, whereas neutrophils and T cells did not significantly differ between sexes (Fig. 5E). Together, these findings indicate that, albeit increased leukocyte influx into the peritoneal cavity in male animals, female animals exhibit a modest trend toward an immunoregulatory local immune response.

### Effects of sex on systemic immune response

**Fig. 6:**
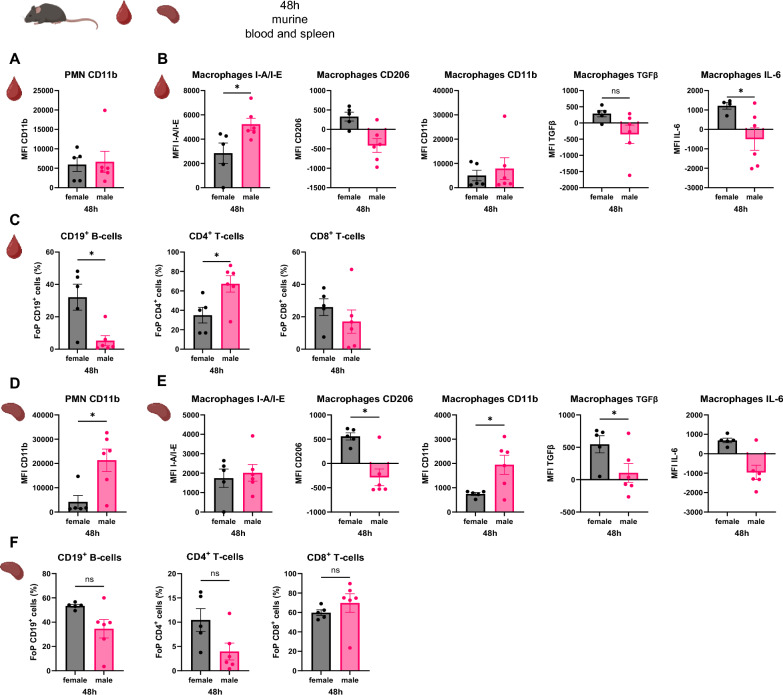
Systemic immune response alteration based on sex*.* Murine PBMC surface markers of (**A**) neutrophils and (**B**) macrophages are displayed as MFI. B and T cell composition (**C**) in the blood, and (**D-F**) in the spleen was assessed. Data are mean ± SEM, *P*-values were determined by Mann–Whitney test (**C** B-cells, **D**, **E** CD206), Welch test (**B-C, E** rest). *P < 0.05. Partially created in BioRender

To evaluate sex-specific systemic immune responses, chemokine concentrations in blood were measured, and immune cell activation as well as T cell subset composition were determined in peripheral blood mononuclear cells (PBMCs) by flow cytometry (Fig. 6). Only slight differences in macrophage function were observed (Fig. 6B), whereas T cell composition differed more noticeably between males and females (Fig. 6C). In splenic immune cells, similar trends were observed for macrophages (Fig. 6E), but not for T cells (Fig. 6F). Similar to the local peritoneal response, the systemic immune response was assessed using a multimodal approach. Minor differences observed did not affect outcome parameters.

### Sex-dependent alterations in hematopoietic stem and progenitor cell compartments during early sepsis

**Fig. 7:**
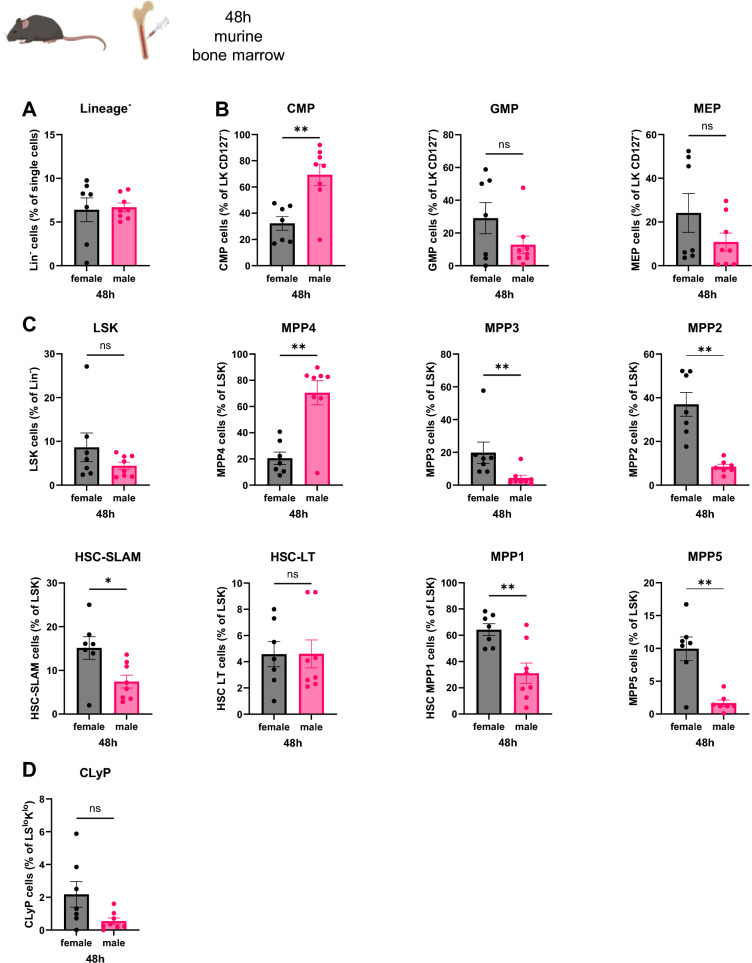
Sex-dependent hematopoietic alterations during early CLP-induced sepsis*.* **A**, Proportions of viable Lin⁻ bone marrow cells in female (black) and male (red) mice after 48 h of CLP sepsis. **B,** Proportions of myeloid progenitors (LK, ckit^low^/sca^low^/CD127⁻) and its myeloid progenitors (CMP), granulocyte-monocyte progenitors (GMP), and megakaryocyte-erythroid progenitors (MEP). **C,** Proportions of hematopoietic stem and progenitor cells (LSK) and their differentiated compartments, including MPP2, MPP3, MPP4, MPP5, SLAM-HSC, MPP1, and LT-HSC populations. **D,** Proportion of common lymphoid progenitors (CLyP, Sca1^low^c-Kit^low^/Flt3^+^/CD127^+^). Data are presented as mean ± SEM. *P*-values were determined using Welch’s test (**B** CMP; **C** MPP2, SLAM-HSC, LT-HSC, MPP1, MPP5; **D**) or Mann–Whitney U test (remaining analyses). *P < 0.05, **P < 0.01. Partially created in BioRender

To determine whether sex-dependent immune responses were accompanied by differences in stress hematopoiesis (Fig. 7), we analyzed hematopoietic stem and progenitor cell subsets within the LSK (Lin⁻Sca-1⁺c-Kit⁺) compartment 48 h after CLP induction. Male mice displayed significantly higher proportions of CMPs (common myeloid progenitor cells) (*P* = 0.025, Fig. 7B) and MPP4 (multipotent progenitor) cells (*P* = 0.0093, Fig. 7C), indicating enhanced myeloid commitment. In contrast, female mice showed significantly higher proportions of total HSCs (*P* = 0.0284), MPP1 (*P* = 0.0036), MPP2 (*P* = 0.0017), and MPP5 (*P* = 0.0031) subsets (Fig. 7C), suggesting broader preservation of immature and multipotent progenitor compartments. The increase in MPP4 and CMPs in males is consistent with a more pronounced emergency myelopoietic response and aligns with the increased peritoneal leukocyte/neutrophil influx observed in male mice. Conversely, enrichment of HSC, MPP1, MPP2, and MPP5 populations in females suggests a less myeloid-skewed hematopoietic response with relative maintenance of early progenitor diversity. Together, these findings indicate that sex-dependent immune responses during early CLP-induced sepsis extend to the bone marrow compartment, with male mice showing stronger myeloid priming and female mice retaining a broader immature HSPC profile.

## Discussion

In this study, we demonstrate that a standardized CLP framework enables reproducible survival and immune readouts across operators and experiments, while allowing systematic identification of biological and environmental sources of variability. Standardization of animal disease models typically includes procedure-specific parameters as well as animal-related variables such as age, sex, body weight, and genotype. In this retrospective analysis, we aimed to go one step further by incorporating variables not immediately apparent to outside observers to assess whether these factors also influence outcomes during the early hyperdynamic phase of CLP-induced sepsis. Interestingly, neither sex, age, body weight, cage density, nor time of day significantly influenced survival following sepsis, and animal sex only subtly affected the immune response. In contrast, the summer season had a measurable and statistically significant effect on survival probability. These findings remained unchanged when analyzed using a multivariate Cox proportional hazards model. Nevertheless, correction for multiple testing would render these results statistically non-significant, and all findings should therefore be interpreted as hypothesis-generating.

The course of sepsis takes different paths depending on the patient’s sex. While incidences in humans between the two sexes do not differ significantly, there are various studies that reported a better outcome in female sepsis patients. [40–42] However, sex was in some cases not an independent predictor but linked to fewer preexisting comorbidities or a different site of infection [43, 44]. Moreover, males have been reported to present higher organ failure parameters [45, 46]. Evidence regarding sex-specific outcomes in the murine CLP sepsis model remains inconsistent. Garcia, Singh [16] reported a trend toward higher mortality in female mice within the first 72 h post-induction, followed by a reversal at later time points. While the majority of studies suggest improved survival in female mice [47–50], others have described the opposite association [51, 52] during CLP and in a LPS model of septic shock.[53] A comprehensive overview is provided in Garcia, Singh [16]. In our study, female mice exhibited a slightly higher mortality in the early phase after sepsis induction; however, this difference did not reach statistical significance. In contrast, a multivariate Cox regression analysis adjusted for multiple covariates indicated a trend toward higher mortality in male mice. Overall, our data do not support a significantly worse outcome in males.

Sex-related differences in sepsis mortality have been hypothesized to depend on hormonal status, as sex hormones are known modulators of immune responses. In murine models, the male sex hormone testosterone has been shown to suppress macrophage chemokine production and alter T cell composition [54]. Sarkar, Mazgaeen [53] reported that, following LPS challenge, female mice produced higher levels of proinflammatory chemokines, whereas male mice exhibited a greater influx of neutrophils. Similarly, Knöferl, Angele [49] observed an impaired macrophage function in male mice after trauma-hemorrhage and subsequent sepsis. Our findings are partially consistent with these observations. Male mice demonstrated increased cellular migration into the peritoneal cavity, although differences in neutrophil recruitment did not reach statistical significance. In contrast, female mice showed higher macrophage-derived IL-6 levels in the blood and a similar trend in peritoneal lavage fluid. Our data indicate distinct sex-specific immune cell responses during sepsis, particularly regarding immune cell migration. However, there was a lesser distinction in immune cell function, and no significant impact on survival was observed.

Although CLP sepsis has been shown to induce profound hematopoietic alterations, including HSPC impairment, emergency myelopoiesis, impaired lymphopoiesis, and bone marrow niche remodeling, sex-dependent hematopoietic responses have not been systematically characterized in this model [18, 19, 55]. Existing CLP studies rarely report HSC and progenitor subsets stratified by sex, leaving the contribution of sex to sepsis-induced hematopoietic remodeling insufficiently defined. In the present study, male animals exhibited a more pronounced myeloid-biased hematopoietic response, whereas female animals retained comparatively broader immature progenitor compartments, suggesting that sex-dependent immune responses during sepsis may already emerge at the level of hematopoietic lineage commitment and emergency myelopoiesis. Despite increasing recognition of hematopoietic remodeling as a relevant component of sepsis pathophysiology, bone marrow and HSPC analyses remain insufficiently standardized in experimental sepsis research and are not included in current MQTiPSS recommendations [8], thereby limiting comparability between studies. Our findings emphasize the need for standardized hematopoietic profiling and sex-stratified reporting of bone marrow and HSPC responses in future CLP studies.

In humans, sepsis incidence and mortality significantly increase with advancing age [42, 44]. However, this relationship is poorly reflected in laboratory animal studies, as most experiments are conducted in young mice. Accordingly, Starr and Saito [56] estimated in 2014 that not even 1% of basic research studies included animals of appropriate age. The few studies that have examined mortality in aged mice, however, consistently report worse survival in older animals [57, 58]. In our study, we included mice aged 10–40 weeks, corresponding to young and middle-aged adults [59]. In our cohort, we did not observe an increased survival in younger animals. However, truly aged mice (commonly defined as approximately 24 months old) were not included. Moreover, the distribution of animals across seasons was unbalanced, with younger mice more frequently undergoing surgery during the summer, which may confound the results. The inclusion of aged mice in sepsis studies should therefore be strongly considered in future work to improve translational relevance and align with the 3Rs principle (replacement, reduction, refinement). However, this approach is associated with practical challenges, including increased housing costs due to extended lifespan and the prolonged time required from breeding to experimental use.

Laboratory mice are generally considered social animals and are therefore typically housed in groups. While housing environment can influence inter alia the need for pain relief after surgery [60] and environmental enrichment has been shown to reduce blood bacterial load following CLP [61], the influence of housing density on animal welfare remains unclear. Reports on aggression, plasma and fecal corticosteroids, food and water intake, and behavioral parameters are ambiguous [38]. The effect of housing density on sepsis in the CLP model has, to our best knowledge, not been addressed. We compared mice housed at low (1–2) versus higher (3–5) densities with respect to survival and observed no differences, including in a multivariate Cox regression model. These results support the notion that cage density may have a limited impact on outcomes in the CLP sepsis model. Nevertheless, given the scarce data in the literature, future studies should consider housing density as a potential variable.

Human sepsis shows a trend toward higher incidence during winter months, particularly due to increased respiratory infections during colder periods. Moreover, the case fatality rate appears to be higher in winter than in summer [62, 63]. In mice, especially when housed under SPF (specific-pathogen-free) conditions, seasonal effects on susceptibility to infections seem unlikely. In laboratory rodents, however, seasonal variations occur despite standardized, controlled conditions [64]. Likewise, in the present study, we observed a higher mortality rate in mice undergoing surgery during the summer months than at other times of year, despite controlled conditions in an SPF animal facility with a 20 °C temperature and a 12-h light cycle. This effect remained significant after adjustment for multiple covariates in a Cox proportional hazards model. Notably, similar observations have been reported before mice undergoing CLP [16, 65], while others reported different seasonal effects [66]. In a colon ascendens stent peritonitis model, higher levels of the proinflammatory cytokine TNF-α were measured during summer and reciprocally higher IL-10 levels during the winter period [65]. Unfortunately, there was a strong correlation between sex and season in this study, such that analyses of the immune response by season are likely confounded by sex (and vice versa), thereby limiting the ability to draw firm conclusions. Garcia, Singh [16] found a slightly higher median temperature and notably higher median humidity in their animal facility, postulating that an altered ambient temperature may have influenced immune response and, thus, survival. Unfortunately, no exact data on facility temperature and humidity over the four years of experiments is available. Suckow and Tirado-Muñiz [67] conclude that external environmental factors underlying seasonal variation in animal experiments are difficult to control and that additional intrinsic factors may also contribute. Therefore, attributing the seasonal variation observed in our study to a single, or even a limited number of variables, is not feasible. We therefore emphasize the importance of precise reporting of the season in which experiments are conducted for reproducibility and, where possible, recommend performing experiments across all seasons.

Nutrition therapy in critically ill patients, especially those affected by sepsis, is difficult due to physiological alterations [68]. In mice, simple calorie restriction compared with ad libitum feeding appeared to increase mortality after CLP sepsis induction [69], whereas a high-fiber diet reduced mortality in another study [70]. In this study, mice were fed a standard maintenance diet ad libitum, while a subset of animals additionally received autoclaved oats placed on the cage floor. These animals showed a non-significant trend toward improved survival probability in the very early phase after sepsis induction, as well as slightly less weight loss after 12 h. However, no differences between groups were observed after 48 h. Supplementation of the diet of laboratory mice with oats has shown positive effects in various inflammatory disease models, such as inflammatory bowel disease [71] and atherosclerosis [72]. The lack of a detectable benefit in our study may be due to the timing, composition, or amount of supplementation, as well as the severity of the model. Taken together, our findings do not support a major impact of oat supplementation on sepsis outcomes under the conditions tested, but dietary factors should still be carefully controlled and reported, as they may act as potential confounders.

Standardization of CLP surgery may fail at the level of the individual experimenter despite rigorous efforts to implement uniform operating protocols and scoring systems. Notably, only a few studies report the number of surgeons involved, and even fewer assess potential inter-operator variability. Garcia, Singh [16], for instance, did not observe differences in outcomes across five surgeons. In our cohort, all procedures were supervised by a single experienced surgeon, while two different scientists assisted during surgeries. In the early phase of the study, the surgeon’s identity was not consistently documented at the individual-animal level. Postoperative scoring was performed by the same teams. Although we observed minor differences in survival during the early postoperative period, these did not reach statistical significance. While our data do not allow for a definitive assessment of experimenter-dependent effects, such influences should not be disregarded. Beyond variability in scoring interpretation and the individual scientists’ experience, the characteristics of the experimenter – such as sex – may also affect outcomes. For example, female rats have been shown to exhibit stronger stress responses when handled by male experimenters compared to female experimenters [73]. Moreover, laboratory animals may respond to subtle human cues, including emotional states [74]. We therefore strongly recommend that future studies systematically document the identity of all personnel involved in surgical procedures and outcome assessment. Such reporting would enable the detection of potential clustering effects attributable to individual experimenters and improve reproducibility.

Sample size in each independent experiment is rarely reported. We observed a 48 h survival of 67.2% in a cohort of 67 mice observed for at least 48 h post-intervention. (n_base_ = 67, p_base_ = 0.672), yielding a 95% confidence interval of 54.6%–78.2% determined by the Clopper-Pearson method. As expected due to random variation, the observed 48 h mortality varied between experiments (Fig. 2D). The probability that the number of surviving animals falls within the previously determined confidence interval in at least 50% of cases for a certain total number of animals is depicted in Table 2, the mathematical calculations can be found in the Supplemental Methods.

**Table 2:** Computational results

m	k_min_	k_max_	*P*(*p*_*new*_ ∈ *CI*)
4	3	3	0.398
5	3	3	0.326
6	4	4	0.329
7	4	5	**0.561**
8	5	6	**0.548**
9	5	7	**0.713**
10	6	7	0.486
11	7	8	0.479
12	8	9	**0.641**

P values > 0.5 are in bold.

We suggest that using a prespecified number of mice per experimental group can help reduce the probability of observing results that deviate substantially from the expected outcome purely by chance. Naturally, increasing the initial sample size ${n}_{\mathrm{b}\mathrm{a}\mathrm{s}\mathrm{e}}$ of the baseline estimate narrows the confidence interval, which in turn increases the number of mice $m$ required in future experiments to achieve the same ≥ 50% probability of falling within the CI. Practically in our study, including 8 wild-type and 8 genetically modified mice per group represents a reasonable compromise between statistical reliability and experimental workload. Based on these calculations, we recommend conducting at least two, and preferably three, independent experiments, each including the calculated sample size.

### Limitations

Several limitations of this study should be acknowledged. First, the 96 h follow-up period was relatively short compared with those of other studies, which reported observation periods of several days or longer. Moreover, most mice were sacrificed after 48 h, as the original study primarily focused on alterations in the immune response during the early hyperdynamic response to the septic stimulus, and hence, the primary outcome in this analysis was 48 h survival. Second, detailed documentation of which experimenter performed surgery on each individual mouse was unavailable; therefore, we could only assess the combined effect of the three experimenters during surgery and postoperative scoring, rather than their individual contributions. Third, most of the mice were transgenic, carrying the loxP site OR the cre recombinase. Influence on the phenotype cannot ultimately be ruled out. Fourth, additional unmeasured factors may have influenced the outcomes. However, including more variables could lead to overfitting of the combined-effects models, particularly given the limited sample size. Finally, we did not apply a global correction for multiple testing, which would likely render the observed sex-specific and procedure-related differences in outcomes and immune function statistically non-significant. Therefore, all results should be interpreted as hypothesis-generating within a multimodal murine sepsis framework.

## Conclusion

Under standardized conditions, the CLP sepsis model in C57BL/6 mice yields reproducible survival outcomes largely independent of common biological and procedural variables, including age, sex, body weight, cage density, timing of surgery, acclimatization, and dietary supplementation. Although sex did not significantly influence survival, the distinct immune responses reported here support the inclusion and separate analysis of both sexes in future studies. In contrast, seasonal variation was associated with altered survival, highlighting environmental factors as relevant confounders. Systematic documentation and reporting of biological, environmental, and procedural variables are essential to improve reproducibility, comparability, and interpretation in preclinical sepsis research.

## Methods

### Murine cecal ligation and puncture (CLP) model

The study was conducted during 2022–2026 in compliance with the local guidelines approved by the Regierung von Oberbayern (ROB-55.2–2532.Vet_02-21–206). Mice were bred at Charles River, Calco, Italy, and subsequently imported into our animal housing facility, where they were housed in a pathogen-free environment maintained at 20 °C with a 12-h light/dark cycle from 6 p.m. to 6 a.m. Animals were kept in Sealsafe® Plus cages (Tecniplast, GM500; 524 cm^2^ floor area) with a maximum of five mice per cage. Maximum cage density was in accordance with EU Directive 2010/63/EU, with ≤ 5 animals per cage for mice weighing 30–40 g and ≤ 4 animals weighing 40–50 g. Mice had ad libitum access to water and to an autoclaved standard maintenance diet (Altromin, #1324SP, 10 mm pelleted), which was additionally placed on the cage floor after sepsis induction to improve food availability. Mouse experiments were blinded by assigning numerical codes to samples. Mice on a C57BL/6 J background carrying the loxP sequence in various genes were crossed with C57BL6 LysM^CreERT2^, Pdgfrb^CreERT2^, Ubc^CreERT2^, and Roza26^CreERT2^ mice (The Jackson Laboratory, IMSR_JAX:032291). In this publication, C57BL6^wt^ mice, C57BL6^loxP/loxP^ CreERT2^−/−^ mice, or C57BL6^−/−^ CreERT2^tg^ mice, hence, phenotypically wildtype, were included. Genotype was confirmed by PCR analysis of tail-tip samples. Most of the mice received daily intraperitoneal injections of Tamoxifen (75 mg/kg body weight; Sigma-Aldrich, #T5648, dissolved in Miglyol, #7660–12-3, sterile-filtered using the Corning® bottle-top vacuum filter, #CLS431153 at a concentration of 20 mg/ml) for five consecutive days, followed by a two-day rest. A few mice received therapeutic antibodies, control antibodies, or PBS injections one day prior to surgery and on the day of the surgery. Of the latter, only animals of the PBS group were included in this analysis. We induced anesthesia using midazolam (5 mg/kg BW), medetomidine (0.5 mg/kg BW), and fentanyl (0.05 mg/kg BW). Vital parameters, including heart rate, oxygen saturation, and temperature, were monitored throughout the procedure (setup in Suppl. Figure 4A). For the CLP procedure, the abdomen was surgically opened via midline laparotomy, the cecum was exteriorized, one-third was ligated and punctured with a 20G needle, and a small amount of fecal content was extruded before returning the cecum to the abdominal cavity as described in [5] (Suppl. Figure 4B-D). After a bi-layer simple interrupted suture of the abdomen (Suppl. Figure 4E), the mice received an intraperitoneal injection of 1 ml prewarmed 0.9% saline and a subcutaneous injection of Buprenorphine 0.1 mg/kg body weight. The surgical wound was treated topically with 2% lidocaine for early postoperative analgesia. Anesthesia was reversed using flumazenil and atipamezole, and mice were returned to clean cages with access to water and food. In later experiments, mice were additionally provided with autoclaved oats placed on the cage floor. Postoperatively, buprenorphine was administered every 6 h; following protocol refinement, it was additionally provided via bacon-flavored Nutra-Gel (Plexx, #9,681,901) at a concentration of 1 mg per 100 g gel. Nutra-Gel was liquefied in a water bath, buprenorphine was injected through the disinfected lid, and the gel was thoroughly mixed by shaking. The Nutra-Gel was provided to the animals in a Nombrero® feeder (Plexx, #13381). To acclimate mice to the taste, Nutra-Gel without buprenorphine was offered for one to two days prior to CLP. Health status was monitored every 6 h. Mice were euthanized at predetermined time points (12, 24, 48, 96 h) or when humane endpoints were reached. Euthanasia was performed using isoflurane and cervical dislocation. Subsequently, peritoneal lavage, spleen, blood, and peritoneum were collected for further analysis as detailed below. Control peritonea and peritoneal lavages were harvested from baseline healthy controls.

### Murine sample processing

Samples from peritoneal lavage, blood, spleen, and bone marrow were processed according to standardized protocols. The spleen was dissociated by passing it through a 70 μm cell strainer (VWR, #732–2758). Tibiae and femurs were cut and placed into perforated 200 µl tubes nested in 1.5 ml Eppendorf tubes. Bone marrow was then centrifuged into the larger Eppendorf tubes. Red blood cells were lysed in samples obtained from spleen, peritoneal lavage, bone marrow, and blood using RBC lysis buffer (BioLegend, #420,301) for 5 min.

### Flow cytometry

Cells were permeabilized with CytoFix/CytoPerm solution (BD, #554,722) and nonspecific binding was blocked with TrueStain fcX (BioLegend, #422,302) in all tissues except bone marrow according to the manufacturer’s protocol. Then, we stained the single cells with antibodies diluted 1:100 as listed in Tables 3, 4 and 5. In the original study, a primary antibody was used in the T-cell and monocyte/macrophage panel depending on the protein of interest. As these proteins are not the focus of the present study, the antibodies are not included in the Methods section. After an overnight incubation, cells were washed and analyzed. Gates were set using fluorescence-minus-one (FMO) controls. We assessed chemokine levels in murine peritoneal lavage at 48 h using the Proinflammatory Chemokine Panel (13-plex) (BioLegend, #740,451). Samples were acquired on a BD LSRFortessa™ flow cytometer, and analysis was performed with FlowJo software (Treestar, V10.10).

**Table 3:** Murine monocyte/macrophage/B-cell antibody panel

Antibody	Fluorophore	Company	Clone	Catalogue number
CD45	BV510	BioLegend	30-F11	103,138
CD11b	BV785	BioLegend	M1/70	101,243
Ly-6G	BV650	BioLegend	1A8	127,641
Ly-6C	AF700	BioLegend	HK1.4	128,024
F4/80	PE-Dazzle594	BioLegend	BM8	123,146
MRC1 (CD206)	Pe-Cy7	BioLegend	C068C2	141,719
FLT-3 (CD135)	BV421	BioLegend	A2F10	135,314
CD19	PE-Fire640	BioLegend	6D5	115,573
I-A/I-E	FITC	BioLegend	M5/114.15.2	107,606
2nd PE Donkey anti-rabbit IgG	PE	BioLegend	Poly4064	406,421
IL-6	APC	BioLegend	MP5-20F3	504,508
IL-10	PerCPCy5	BioLegend	JES5-16E3	505,027
IFN-g	BV710	BioLegend	XMG1.2	505,836
TGF-b	AF750	Novus Biologicals	TGFB/510	NBP3-08739AF750

**Table 4:** Murine T-cell antibody panel

Antibody	Fluorophore	Company	Clone	Catalogue number
CD45	BV510	BioLegend	30-F11	103,138
CD3	APC-Fire 750	BioLegend	17A2	100,248
CD4	PerCP-Cy5.5	BioLegend	GK1.5	100,434
CD8a	BV785	BioLegend	53–6.7	100,750
CD62L	BV650	BioLegend	MEL-14	104,453
NK1.1 (CD161)	BV711	BioLegend	PK136	108,745
CX3CR1	PE-Cy7	BioLegend	SA011F11	149,016
Neuropilin-1	BV421	BioLegend	3E12	145,209
GATA-3	APC	BioLegend	10A23	653,806
T-bet	PE-Dazzle 594	BioLegend	4B10	644,828
2nd PE Donkey anti-rabbit IgG	PE	BioLegend	Poly4064	406,421
FoxP3	AF700	BioLegend	MF-14	126,422

**Table 5:** Murine hematopoiesis antibody panel

Antibody	Fluorophore	Company	Clone	Catalogue number
Lineage	FITC	BioLegend	145-2C11; RB6-8C5; RA3-6B2; Ter-119; M1/70	133,302
Ly-6A/Ly-6E	PE	BioLegend	QA17A36	163,803
CD117	APC-Cy7	BioLegend	ACK2	135,140
CD150	BV711	BioLegend	TC15-12F12.2	115,941
CD34	PE-Cy5	BioLegend	MEC14.7	119,311
CD69	PE-Cy7	BioLegend	H1.2F3	104,511
Flt-3	BV421	BioLegend	A2F10	135,314
CD127	APC	BioLegend	A7R34	135,012
CD16/32	PE-Dazzle594	BioLegend	S17011E	156,615
CD48	BV785	BioLegend	HM48-1	103,449

### Histological preparation and analysis

Fixed peritoneal samples were embedded in paraffin, sectioned at a thickness of 4 μm, and stained with hematoxylin and eosin. Slides were scanned using a Leica Aperio AT2 scanner, and digital images were analyzed using Aperio ImageScope and ImageJ2 (v2.14). Cell density (cells per area) and submesothelial thickness were measured at multiple sites per sample, and mean values were calculated.

### Gating Strategy

Peritoneal lavage fluid, lysed whole blood, and spleen single-cell suspensions were analyzed using a sequential gating strategy to identify major leukocyte populations. Initially, leukocytes were selected based on forward scatter (FSC) and side scatter (SSC) parameters to exclude debris and aggregates (Suppl. Figure 5A). Doublets and multiplets were then removed using FSC-H vs. FSC-A gating to ensure single-cell analysis (Suppl. Figure 5B). Non-viable cells were excluded using a viability dye staining (Suppl. Figure 5C. A dead control is depicted on the right-hand side). Leukocytes were subsequently defined as CD45⁺ cells (Suppl. Figure 5D). Within the CD45⁺ population, further sub-gating was performed to delineate specific cell types. Cells were classified based on Ly6-C and Ly6-G expression into Ly6-C^high^ and Ly6-C^low^ monocytes, as well as Ly6-G⁺ polymorphonuclear neutrophils (PMNs) (Suppl. Figure 5E). F4/80⁺ macrophages and CD135⁺ cells were also identified within this compartment (Suppl. Figure 5F). CD19⁺ cells were gated to define B cells (Suppl. Figure 5G). In a separate antibody panel, CD3⁺ T cells were identified and further subdivided into CD4⁺ T helper (Th) cells and CD8⁺ cytotoxic T (Tc) cells (Suppl. Figure 5H-I). Each gating step refined cell population identification for downstream analyses.

Bone marrow single cells were initially gated based on FSC and SSC characteristics to exclude debris (Suppl. Figure 6A), followed by doublet and multiplet discrimination applying FSC-H vs FSC-A (Suppl. Figure 6B). Within the viable lineage-negative compartment (lin-) (Suppl. Figure 6C), common lymphoid progenitors (CLyPs, Sca1^low^c-Kit^low^/Flt3^+^/CD127^+^), myeloid progenitors (LK, Sca1^−^ c-Kit^+^) and hematopoietic stem and progenitor cells (LSK, Sca1^+^ c-Kit^+^) were identified as previously described (Suppl. Figure 6D) [75]. MPP4 cells were defined as Flk2^+^ (CD135^+^) LSK cells. Within the Flk2^−^ LSK compartment, MPP2, MPP3, and MPP5 cells were identified based on CD48 and CD150 expression. SLAM-HSCs (Signaling Lymphocytic Activation Molecule-HSCs) were defined as Flk2^−^CD48^−^CD150^+^ LSK cells and further subdivided into CD34^+^ MPP1 and CD34^−^ LT-HSC (long-term hematopoietic stem cells) populations (Suppl. Figure 6E-J). Representative gating strategies are shown in Supplementary Fig. 6.

### Statistical analysis

Data were tested for normality using the Shapiro–Wilk test. For normally distributed data, comparisons between two groups were performed using the Welch test, and comparisons among more than two groups were conducted using one-way ANOVA (Analysis of Variance) followed by Tukey’s post hoc test. For non-normally distributed data, the Mann–Whitney U test was used for comparisons between two groups, and the Kruskal–Wallis test followed by Dunn’s test was applied for comparisons among more than two groups. Categorical variables were compared using the chi-square (χ^2^) test. In cases where expected cell frequencies were < 5, Fisher’s exact test was used instead. A 95% confidence interval for the observed proportion of surviving animals was calculated using the exact Clopper-Pearson method. Correlation analyses were performed using Spearman’s rank correlation coefficient. Linear regression lines in scatter plots were fitted using the ordinary least squares method. Differences between regression lines were assessed using analysis of covariance (ANCOVA). Survival differences between two groups were evaluated using the Kaplan–Meier estimator with the log-rank test. Hazard ratios for individual variables were calculated using a univariate Cox proportional hazards model. The influence of multiple variables on survival was assessed using a multivariate Cox proportional hazards model. All statistical tests were two-sided. A *P*-value < 0.05 was considered statistically significant. Graphical data are presented as mean ± standard error of the mean (SEM). Tabular data are presented as mean ± standard deviation (SD) for normally distributed variables, or as median and interquartile range (IQR) for non-normally distributed variables. No general correction for multiple testing was applied due to the exploratory nature of this study. Statistical analyses were performed using IBM SPSS Statistics (version 29.0.0.0) and GraphPad Prism (version 11.0.2).

## Supplementary Information

Supplementary Material 1: Supplemental Methods

Supplementary Material 2: Supplemental Fig. 1**.** *The CLP sepsis model.* **S1A**, Mice were operated in a dedicated surgical setup equipped with pulse oximetry and continuous temperature monitoring. **S1B**, After induction of anesthesia, the mouse was placed on the operating table, and the abdomen was shaved and thoroughly disinfected. **S1C**, The abdomen was opened via a midline laparotomy, and the cecum was carefully exteriorized. **S1D**, After ligation of the distal third, the cecum was punctured with a 20G needle, and a small amount of fecal content was gently extruded. **S1E**, The abdomen was closed using a two-layer, single-stitch suture.

Supplementary Material 3: Supplemental Fig. 2**. S2A,** Scatter plot displays age in weeks and baseline body weight in grams in female (black) and male (red) mice. **S2B**, Scatter plot displays cage density and baseline body weight in female (black) and male (red) mice. Spearman correlation coefficient ρ (**S2A**), correlation coefficient β in a multiple linear regression model (**S2B**).

Supplementary Material 4: Supplemental Fig. 3**. S3A,** Stacked bars plot of mice divided into two age groups and sex. **S3B**, Stacked bars plot of mice divided into two age groups and the season of surgery. **S3C**, Kaplan–Meier plot of the survival in mice operated on in the summer or the remaining seasons. **S3D**, Violin plot of the weight loss in mice receiving additional oats (brown) or none (black). **S3E**, Kaplan–Meier plot of the survival in mice injected with tamoxifen (dark blue) or PBS (light blue) prior to surgery. **S3F**, total cell counts within the peritoneal lavage at 48 h hours after sepsis induction in mice injected with tamoxifen (dark blue) or PBS (light blue). Violin plots in **S3D** with median and Kernel Density. Chi-square test (**S3A-B**), survival was estimated using the Kaplan–Meier method, statistical significance refers to the log-rank test, hazard ratios were derived from univariate Cox proportional hazards regression (**S3C, S3E**), Welch test (**S3D**), Mann–Whitney Test (**S3F**). **P < 0.01, ****P < 0.0001. Partially created in BioRender.

Supplementary Material 5: Supplemental Fig. 4. Total cell counts and chemokines within the peritoneal lavage after 48 h of sepsis in baseline healthy control (cyan), mice operated on in the summer (lavender), and during the rest of the year (blue). Data are mean ± SEM. Mann–Whitney Test. **P < 0.01. Partially created in BioRender.

Supplementary Material 6: Supplemental Fig. 5. *Gating Strategy Leukocytes.* Single cell suspensions were subjected to sequential gating to identify major leukocyte populations. **A**, Gate based on forward scatter (FSC) and side scatter (SSC) properties to exclude debris and aggregates. **B**, Doublets and multiplets were excluded. **C**, Viability dye staining allowed exclusion of non-viable cells. The right-hand image shows dead control. **D**, Identification of leukocytes. Then, either **E**, high and low Ly6-C monocytes and Ly6-G + polymorphonuclear neutrophils (PMNs), or **F**, F4/80 + macrophages and CD135 + cells, or **G**, CD19 + cells. In a second antibody panel, downstream of CD45 + , **H**, CD3 + T cells, differentiated into **I**, CD4 + Th cells and CD8 + Tc cells.

Supplementay Material 7: Supplemental Fig. 6. *Gating Strategy Hematopoiesis.* Single cell suspensions were subjected to sequential gating to identify major hematopoietic cell populations. **A**, Gate based on forward scatter (FSC) and side scatter (SSC) properties to exclude debris and aggregates. **B**, Doublets and multiplets were excluded. **C**, Viability dye staining allowed exclusion of non-viable cells, and lineage negative (lin^−^) cells were analyzed for **D**, cKit (CD117) and Sca1 (Ly-6A/Ly-6E) expression. **E&F**, Within the Lin^−^cKit^+^Ly6A^−^- (LK) population, CD127^−^ myeloid-committed progenitors (CMP, GMP, MEP) were identified. **G-I**, Within the Lin^−^cKit^+^Ly6A^+^ (LSK) population, we identified early (MPP1 and MPP5) and lineage-biased (MPP2/MPP3/MPP4) multipotent progenitors. **J**, Within the Lin^−^cKit^low^Ly6A^low^ gated cells, we identified the common lymphoid progenitor (CLyP).

## Data Availability

The datasets used and/or analyzed during the current study are available from the corresponding author on reasonable request.

## Abbreviations

3Rs: Replacement, reduction, refinement
ANCOVA: Analysis of covariance
ANOVA: Analysis of variance
BW: Body weight
CI: Confidence interval
CLP: Cecal ligation and puncture
CLyP: Common lymphoid progenitor (commonly abbreviated as CLP; renamed CLyP herein to avoid ambiguity)
CMP: Common myeloid progenitor
FSC: Forward scatter
GMP: Granulo-macrophagic progenitor
HR: Hazard ratio
HSC: Hematopoietic stem cell
HSPC: Hematopoietic stem and progenitor cell
IQR: Interquartile range
LSK: Lin⁻Sca-1⁺c-Kit⁺
LT-HSC: Long-term hematopoietic stem cells
MEP: Megakaryocytic erythroid progenitors
MPP: Multipotent progenitor
PBMCs: Peripheral blood mononuclear cells
SD: Standard deviation
SLAM: Signaling lymphocytic activation molecule
SSC: Sideward scatter
SPF: Specific-pathogen-free
