# Supplementary material for: Experimental robustness and reproducibility of the murine cecal ligation and puncture sepsis model
Source: Intensive Care Med Exp. 2026 Jul 14;14:91. doi: 10.1186/s40635-026-00950-0 (PMC13369109; doi:10.1186/s40635-026-00950-0)
Supplement: Supplementary file 1 — Supplementary Material 1: Supplemental Methods [file 40635_2026_950_MOESM1_ESM.docx]

**Supplemental Methods**

For planning future experiments, we aimed to determine how many mice of each genotype ($m$) would be required in a single experiment so that the number of surviving animals $x$ falls within the previously determined 95% confidence interval in at least 50% of cases for the predetermined observation period of 48 h, treating the previously observed 48 h mortality as the true underlying event probability. That is, $P (\frac{x}{m}\in\left[ 0.546;0.782 \right]\geq0.50$, hereafter referred to as *p*_new_. The minimal $m$ can be found iteratively by increasing $m$ in steps of 1. For a given $m$, the lower and upper limits for the number of surviving animals are $k_{min}=\left\lceil m\times0.546 \right\rceil$ and $k_{max}=\left\lfloor m\times0.782 \right\rfloor$ (the lower limit must be rounded up to the nearest integer, while the upper limit must be rounded down to the nearest integer). The probability that the observed survival proportion lies within the CI is $P\left( p_{new}\in CI \right)=P(kmin\leq X\leq kmax)=\sum_{k=kmin}^{kmax} P\left( x=k \right)=\sum_{k=min}^{kmax} \binom{m}{k}\times{p_{base}}^{k}\times{(1-p_{base})}^{m-k}.$ Results for $m\in\left\{ 4;12 \right\}$ can be found in table 2. Note that for small values of *m*, the results are not robust, as the width of the calculated confidence intervals varies substantially.
