# Supplementary figures and images for: Experimental robustness and reproducibility of the murine cecal ligation and puncture sepsis model
Source: Intensive Care Med Exp. 2026 Jul 14;14:91. doi: 10.1186/s40635-026-00950-0 (PMC13369109; doi:10.1186/s40635-026-00950-0)

**S1A**

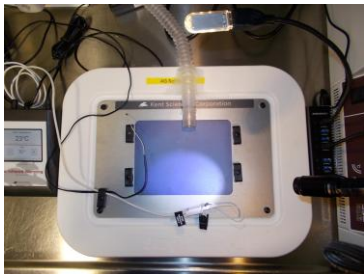

**B**

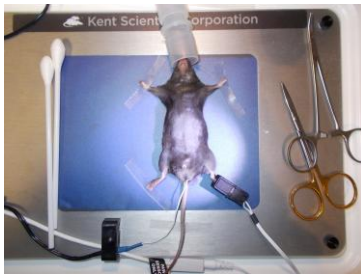

**C**

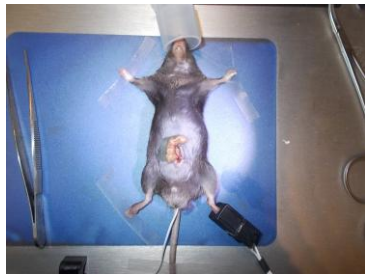

**D**

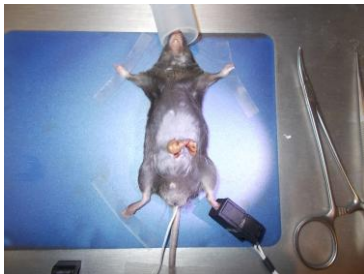

**E**

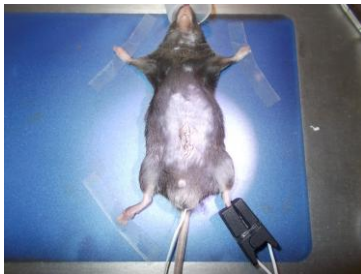

Supplement: Supplementary file 2 — Supplementary Material 2: Supplemental Fig. 1. The CLP sepsis model. S1A, Mice were operated in a dedicated surgical setup equipped with pulse oximetry and continuous temperature monitoring. S1B, After induction of anesthesia, the mouse was placed on the operating table, and the abdomen was shaved and thoroughly disinfected. S1C, The abdomen was opened via a midline laparotomy, and the cecum was carefully exteriorized. S1D, After ligation of the distal third, the cecum was punctured with a 20G needle, and a small amount of fecal content was gently extruded. S1E, The abdomen was closed using a two-layer, single-stitch suture. [file 40635_2026_950_MOESM2_ESM.pdf]

## S2A

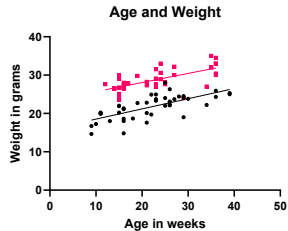

## S2B

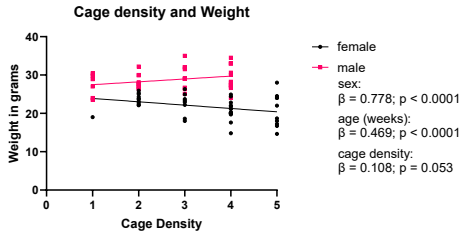

Supplement: Supplementary file 3 — Supplementary Material 3: Supplemental Fig. 2. S2A, Scatter plot displays age in weeks and baseline body weight in grams in female (black) and male (red) mice. S2B, Scatter plot displays cage density and baseline body weight in female (black) and male (red) mice. Spearman correlation coefficient ρ (S2A), correlation coefficient β in a multiple linear regression model (S2B). [file 40635_2026_950_MOESM3_ESM.pdf]

### S3A

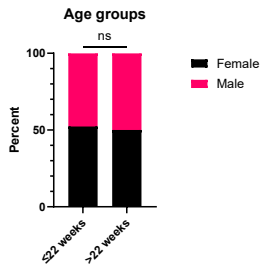

### S3B

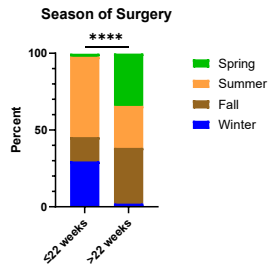

### S3C

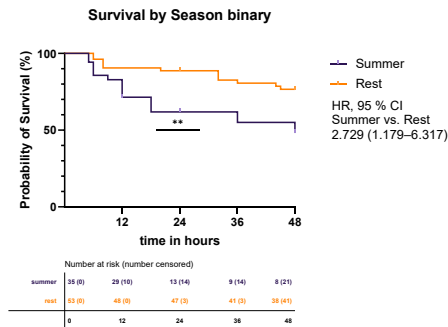

### S3D

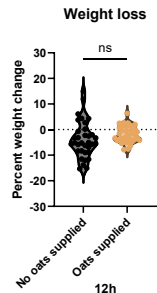

### S3E

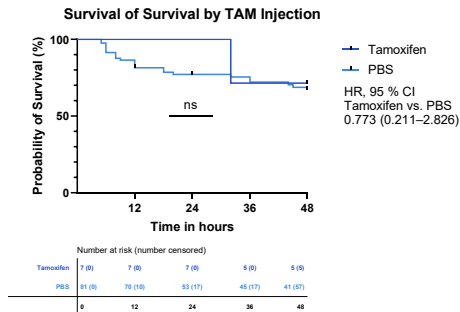

### S3F

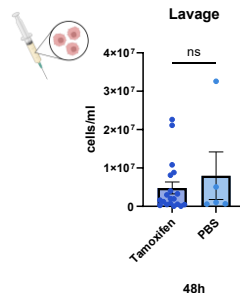

Supplement: Supplementary file 4 — Supplementary Material 4: Supplemental Fig. 3. S3A, Stacked bars plot of mice divided into two age groups and sex. S3B, Stacked bars plot of mice divided into two age groups and the season of surgery. S3C, Kaplan–Meier plot of the survival in mice operated on in the summer or the remaining seasons. S3D, Violin plot of the weight loss in mice receiving additional oats (brown) or none (black). S3E, Kaplan–Meier plot of the survival in mice injected with tamoxifen (dark blue) or PBS (light blue) prior to surgery. S3F, total cell counts within the peritoneal lavage at 48 h hours after sepsis induction in mice injected with tamoxifen (dark blue) or PBS (light blue). Violin plots in S3D with median and Kernel Density. Chi-square test (S3A-B), survival was estimated using the Kaplan–Meier method, statistical significance refers to the log-rank test, hazard ratios were derived from univariate Cox proportional hazards regression (S3C, S3E), Welch test (S3D), Mann–Whitney Test (S3F). **P < 0.01, ****P < 0.0001. Partially created in BioRender. [file 40635_2026_950_MOESM4_ESM.pdf]

S4

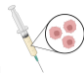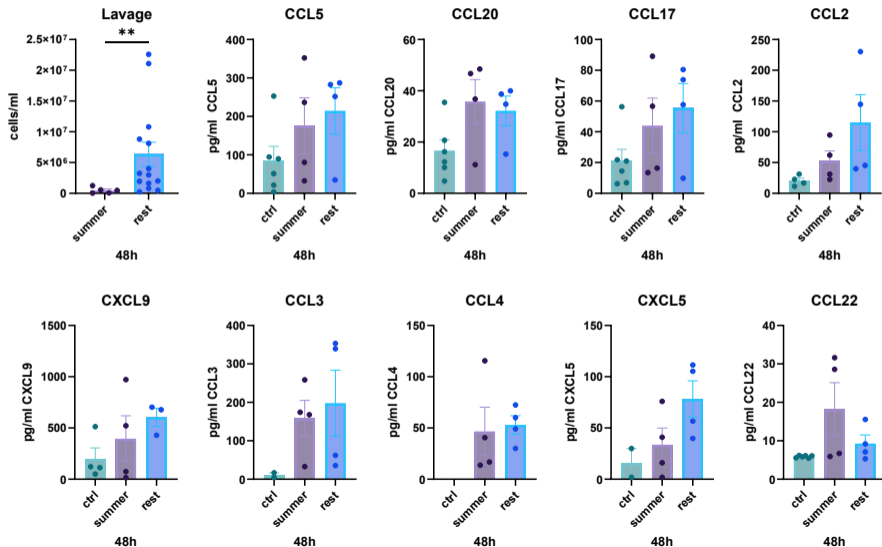

Supplement: Supplementary file 5 — Supplementary Material 5: Supplemental Fig. 4. Total cell counts and chemokines within the peritoneal lavage after 48 h of sepsis in baseline healthy control (cyan), mice operated on in the summer (lavender), and during the rest of the year (blue). Data are mean ± SEM. Mann–Whitney Test. **P < 0.01. Partially created in BioRender. [file 40635_2026_950_MOESM5_ESM.pdf]

**S5A**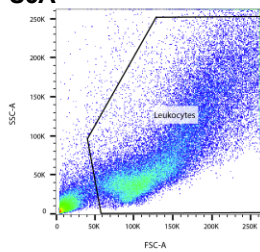**B**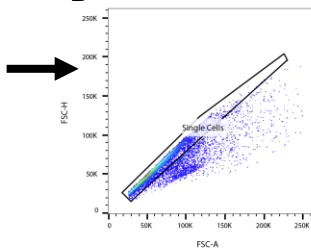**C**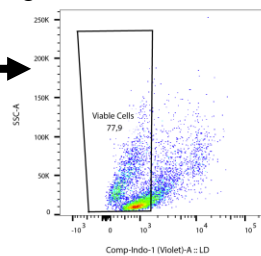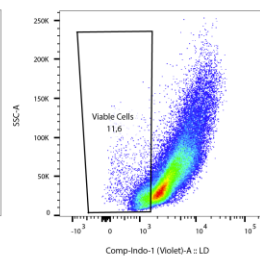**H**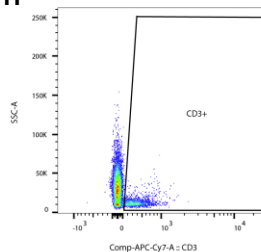**D**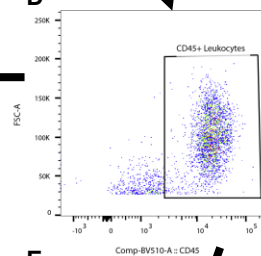**G**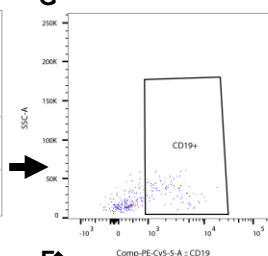**I**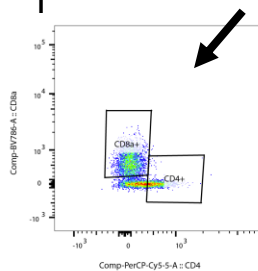**E**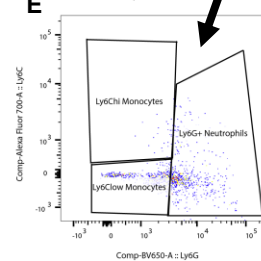**F**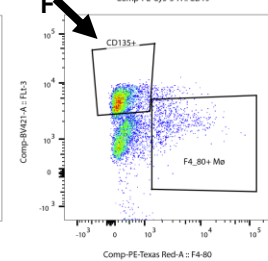

Supplement: Supplementary file 6 — Supplementary Material 6: Supplemental Fig. 5. Gating Strategy Leukocytes. Single cell suspensions were subjected to sequential gating to identify major leukocyte populations. A, Gate based on forward scatter (FSC) and side scatter (SSC) properties to exclude debris and aggregates. B, Doublets and multiplets were excluded. C, Viability dye staining allowed exclusion of non-viable cells. The right-hand image shows dead control. D, Identification of leukocytes. Then, either E, high and low Ly6-C monocytes and Ly6-G + polymorphonuclear neutrophils (PMNs), or F, F4/80 + macrophages and CD135 + cells, or G, CD19 + cells. In a second antibody panel, downstream of CD45 + , H, CD3 + T cells, differentiated into I, CD4 + Th cells and CD8 + Tc cells. [file 40635_2026_950_MOESM6_ESM.pdf]

**S6A**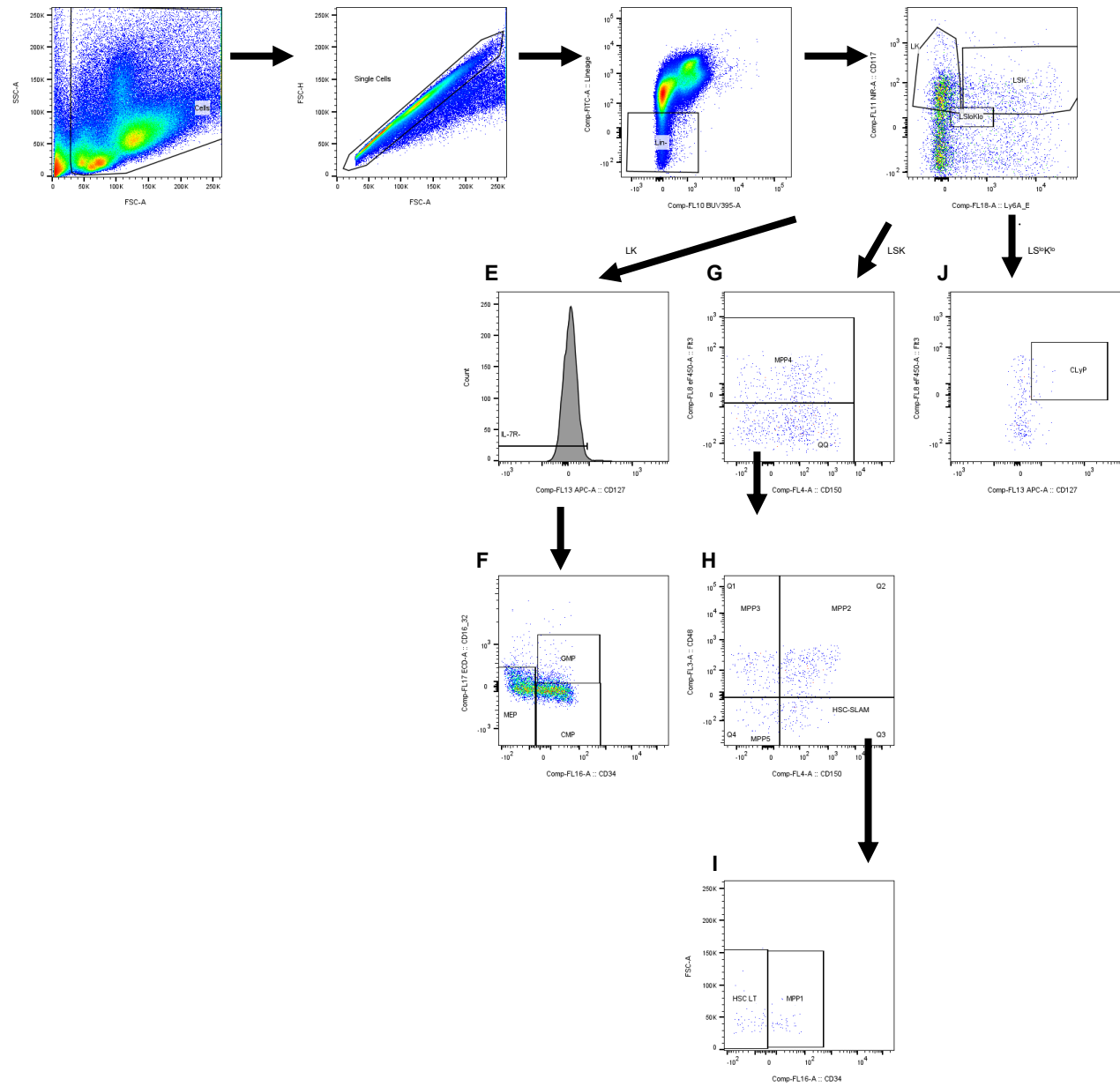

Supplement: Supplementary file 7 — Supplementay Material 7: Supplemental Fig. 6. Gating Strategy Hematopoiesis. Single cell suspensions were subjected to sequential gating to identify major hematopoietic cell populations. A, Gate based on forward scatter (FSC) and side scatter (SSC) properties to exclude debris and aggregates. B, Doublets and multiplets were excluded. C, Viability dye staining allowed exclusion of non-viable cells, and lineage negative (lin−) cells were analyzed for D, cKit (CD117) and Sca1 (Ly-6A/Ly-6E) expression. E&F, Within the Lin−cKit+Ly6A−- (LK) population, CD127− myeloid-committed progenitors (CMP, GMP, MEP) were identified. G-I, Within the Lin−cKit+Ly6A+ (LSK) population, we identified early (MPP1 and MPP5) and lineage-biased (MPP2/MPP3/MPP4) multipotent progenitors. J, Within the Lin−cKitlowLy6Alow gated cells, we identified the common lymphoid progenitor (CLyP). [file 40635_2026_950_MOESM7_ESM.pdf]
